# Economic Impact of Dementia by Disease Severity: Exploring the Relationship between Stage of Dementia and Cost of Care in Taiwan

**DOI:** 10.1371/journal.pone.0148779

**Published:** 2016-02-09

**Authors:** Li-Jung Elizabeth Ku, Ming-Chyi Pai, Pei-Yu Shih

**Affiliations:** 1 Department of Public Health, National Cheng Kung University Hospital, College of Medicine, National Cheng Kung University, Tainan, Taiwan; 2 Division of Behavioral Neurology, Department of Neurology, National Cheng Kung University Hospital, College of Medicine, National Cheng Kung University, Tainan, Taiwan; 3 Alzheimer’s Disease Research Center, National Cheng Kung University Hospital, Tainan, Taiwan; University Of São Paulo, BRAZIL

## Abstract

**Objective:**

Given the shortage of cost-of-illness studies in dementia outside of the Western population, the current study estimated the annual cost of dementia in Taiwan and assessed whether different categories of care costs vary by severity using multiple disease-severity measures.

**Methods:**

This study included 231 dementia patient–caregiver dyads in a dementia clinic at a national university hospital in southern Taiwan. Three disease measures including cognitive, functional, and behavioral disturbances were obtained from patients based on medical history. A societal perspective was used to estimate the total costs of dementia according to three cost sub-categories. The association between dementia severity and cost of care was examined through bivariate and multivariate analyses.

**Results:**

Total costs of care for moderate dementia patient were 1.4 times the costs for mild dementia and doubled from mild to severe dementia among our community-dwelling dementia sample. Multivariate analysis indicated that functional declines had a greater impact on all cost outcomes as compared to behavioral disturbance, which showed no impact on any costs. Informal care costs accounted for the greatest share in total cost of care for both mild (42%) and severe (43%) dementia patients.

**Conclusions:**

Since the total costs of dementia increased with severity, providing care to delay disease progression, with a focus on maintaining patient physical function, may reduce the overall cost of dementia. The greater contribution of informal care to total costs as opposed to social care also suggests a need for more publicly-funded long-term care services to assist family caregivers of dementia patients in Taiwan.

## Introduction

Cost-of-illness (COI) studies provide estimates about the economic impact of diseases and offer comprehensive data to assist decision makers for purposes of planning and financing of health systems [1]. As a degenerative disease with an average duration of 4 to 8 years of survival after diagnosis [2], it is important to understand how costs evolve over the course of the disease [3]. Considering the latest estimate of the global societal costs of dementia to be US$818 billion, or 1.09% of the worldwide Gross Domestic Product (GDP), dementia poses great challenge not only for the patients and their families, but also for health care systems around the world [4]. In the last two decades, research on the COI of dementia has expanded, leading to several systematic reviews focusing on costs of dementia and disease severity [3, 5, 6]. In all of these reviews, disease stage was an important determinant of costs of dementia, and the costs were found to increase with dementia severity. Despite these common findings, large variations were found between various cost estimates due to setting and cultural characteristics [6]. For instance, cultural traditions in East Asia often make adult children reluctant to send their parents with dementia to nursing homes; therefore, the utilization of formal versus informal care can be influenced by culture and ultimately affects COI [7]. It is thus important for each nation to have country-specific data on cost of care at different stages of dementia for health policy planning [8].

Currently, most published COI studies of dementia have been conducted in North America or Europe, as only 4 out of a total of 84 studies included in those three systematic reviews were done in East Asian countries. Given the shortage of COI studies of dementia outside the Western population [9], this article is aimed at investigating the relationship between dementia severity and cost of care using data from Taiwan in order to increase the diversity of the literature.

A review of past epidemiological studies showed the prevalence of dementia in Taiwan among the elderly to be between 1.7% and 4.3%, with Alzheimer’s disease being the most common type of dementia [10]. The latest nationwide survey in Taiwan found that the age-adjusted prevalence of dementia among those 65 years and above was 4.79% [11]. Although the number of people with dementia is clearly on the rise, there have only been a few economic studies about dementia in Taiwan. The first estimate of the COI of dementia in Taiwan was published in 2002, and the figure was between NT$310,018 to 710,737 per patient per year [12]. In 2010, a study provided an updated estimate of $462,700 for the cost of home care [13]. However, as the later study focused on comparison of costs between home-based versus institutional care, it did not report these costs of care according to dementia severity. Therefore, the first aim of the current study is to calculate the annual cost of care for community-based dementia patients in Taiwan and the second aim is to estimate the distribution of different cost categories in proportion to the total cost of dementia. In order to make our cost estimates comparable internationally, we adopted the COI methodology outlined in the 2010 World Alzheimer Report (WAR) to examine three sub-categories of total care costs: direct medical, direct social care, and informal care costs [1].

In addition to providing an updated COI estimate for our sample in Taiwan, this study is also an attempt to address limitations in previous COI studies by including multiple measures instead of a single measure of disease severity [14]. While cognitive status, functional limitation, and behavioral disturbance are all correlated with cost of care, independent associations with costs have been noted for different measures [5]. One study including all three measures found that cognitive status had a greater impact on informal costs while functional declines had a greater impact on social care costs more than medical costs [15]. The third aim of our study is to conduct analyses with multiple disease-severity measures to assess whether different care cost categories vary by dementia severity.

## Materials and Methods

### Study design

We recruited 286 dementia patient–caregiver dyads in a dementia clinic at a national university hospital in southern Taiwan from November of 2013 to April of 2015. We included patients aged ≥65 years with dementia diagnosed by a senior behavioral neurologist (MCP) according to the Diagnostic and Statistical Manual of Mental Disorders, Fourth Edition (DSM-IV) criteria [16]. Dementia subtypes included mostly Alzheimer’s disease (n = 191) as well as dementia with Lewy bodies (DLB) (n = 9), vascular dementia (n = 5) and other unspecified types. Inclusion criteria required that dementia patients had been living in the community and had an informal caregiver. The caregiver of the dementia patient was required to be a family member, ≥18 years of age, who was fluent in either Mandarin or Taiwanese. We excluded paid caregivers or those who had been a caregiver for less than 12 months. Subjects were excluded from this cost analysis if they had younger-onset dementia (n = 31), if living in an institution (n = 8), or if they did not consent to linkage to medical claims data (n = 10). After removing 4 withdrawals and 2 incomplete responses, our final sample included 231 community-dwelling patient–caregiver dyads.

### Study Procedures

Ethics approval was obtained from the National Cheng Kung University Hospital (NCKUH) Institutional Review Board for the Protection of Human Subjects (IRB No: B-ER-102-173). Patients and caregivers were recruited from the NCKUH dementia clinic and written consent for the study was obtained from both the caregivers and the patients. For cognitively impaired patients who could not provide their signatures, proxy consent was obtained from their family caregiver.

Subsequent to the informed consent procedure, telephone interviews with caregivers were conducted by two research assistants who were trained in questionnaire assessments and data entry following the interviews.

### Measures

#### Patients’ measures

Demographic data, including age, gender, and years of education, were collected from both patients and their caregivers. Three clinical measures of disease severity were obtained from the patients based on their history or relevant examinations. Cognitive status was determined with either the Clinical Dementia Rating Scale (CDR) [17] or the Chinese version of the Mini-Mental State Examination (MMSE) [18], depending on which of these was on the patient’s medical record. A CDR global score of 1, 2, and 3 indicates mid, moderate, or severe dementia respectively. If no CDR scores but only MMSE scores were available, we categorized subjects with an MMSE score above 15 as mild dementia, an MMSE score from 10 to 14 as moderate dementia, and those with an MMSE score below 10 as severe dementia according to Taiwan’s National Health Insurance (NHI) reimbursement rule for dementia drugs [19]. Functional status was measured with the Katz Activities of Daily Living (ADL) scale to assess whether the patient was dependent on the following five ADLs: bathing, dressing, toileting, transferring, continence, and feeding [20, 21]. The total ADL score may range from 0 to 100, with higher scores indicating less need for support. The Neuropsychiatric Inventory (NPI) was used to estimate both the severity and frequency of a wide range of behavioral reactions including agitation, depression, and disinhibition [22]. The total NPI score may range from 0 to 120, where higher scores indicate greater impairment.

#### Cost estimates

We collected data on socioeconomic status and household composition from caregivers using a structured questionnaire. This study takes a societal perspective to calculate four cost outcomes, including total costs and three cost sub-categories. First, medical costs included costs of inpatient and outpatient visits, medication use covered by Taiwan’s NHI, and medical expenditures paid out-of-pocket. Medical costs were obtained from linkage to medical claims records for the 12 months prior to the survey. Secondly, social care costs included costs associated with the use of adult daycare, respite care, home care, paid domestic help, and transportation costs. Third, informal costs were valued by the level of contribution to patient care by the caregiver. We assessed care hours based on assistance with (1) basic ADL, (2) instrumental ADL (IADL), and (3) supervision provided by the informal caregiver for the person with dementia in the week prior to the interview. Informal care hours were collected using the Resource Utilization in Dementia (RUD) instrument, where its measurement validity in community-living persons with dementia has been established [23].

Two methods are frequently used for calculating the cost of informal care, namely, the “opportunity cost” and the “replacement cost” approaches [1]. In this study, the opportunity cost approach was selected for our base-case estimates as recommended by the WAR 2010 [1], valuing informal care by the average hourly wage rates in Taiwan stratified by gender and education level. We separated caregivers according to Taiwan’s official retirement age of 65 and only estimated opportunity costs of informal care for those caregivers aged below 65. However, in a sensitivity analysis, we estimated informal care costs using the replacement costs approach in which the mandated wage rates of NT$200/hour (US$6.7/hour) for home care aides were assumed for all caregiving hours [24]. We did not include supervision hours in valuing informal care but rather reported them separately following the WAR 2010 method.

Total costs per annum per person with dementia were calculated by adding medical, social, and informal care costs as valued by the opportunity cost approach. In addition to the base-case estimate, informal care costs valued by the replacement cost approach resulted in an upper bound estimate of total costs. Because our cost data spanned from 2013 to 2014, we inflated all costs to the 2014 values using the Consumer Price Index and reported all costs in 2014 New Taiwan dollars (NTD). The average exchange rates in 2014 were 1 USD = 30.37 NTD and 1NTD = 14.97 US dollars using purchasing power parity (PPP) conversion rates [25], respectively.

#### Statistical analysis

We compared different categories of care costs according to dementia severity using nonparametric Kruskal-Wallis tests since the cost variables were highly skewed. For the multivariate cost analyses, we employed a generalized linear model (GLM) assuming a log link and a Poisson family of error distribution after running a modified Park’s test [26]. In addition to disease severity measures, the GLM analysis also accounted for other patient and caregiver characteristics, including the use of any formal services and the caregivers’ education and economic status. Given the nonlinear nature of our statistical model, the log coefficients do not provide a straightforward interpretation of the substantive influence of predictors [27]. Consequently, marginal estimates were used to illustrate the predicted differences in care costs by disease severity, holding all other variables unchanged. A marginal estimation was carried out using the margins command in Stata version 12 (Stata Corp., College Station, Texas).

## Results

Table 1 shows the demographic and disease characteristics of the patients and their caregivers. The average age of the patients was 80 years, and they had been diagnosed with dementia, on average, 4.6 years prior to inclusion in the study. Our sample consisted of 102 mild, 88 moderate, and 41 severe dementia patients based on the CDR or MMSE scores. The caregivers interviewed were mostly middle-aged (mean age 61 ± 13.2 years), with a majority being women (60%). The majority of the caregivers were highly educated (70% had finished senior high school or college) compared to their care recipients, who had received less education. Combining adult children and children-in-law caregivers together, they accounted for 60% as opposed to 38% of spousal caregivers. In a typical week, these caregivers spent 6.4 hours in assisting dementia patients with basic ADLs and 15 hours with IADLs.

**Table 1:** Characteristics of dementia patients and their caregivers (N = 231).

	N	(%)	Mean	SD
***Patient characteristics***				
Age, years		-	80.0	6.9
Gender (% female)	138	(60%)	-	
Years since diagnosis		-	4.6	3.3
Number of children		-	5.6	2.1
**Education**				
No education	57	(25%)	-	
Elementary school	108	(47%)	-	
Junior high school or above	66	(28%)	-	
ADL score (range: 0–100)			69.1	33.4
NPI score (range: 0–120)			18.0	19.3
**Dementia severity**				
Mild	102	(44%)	-	
Moderate	88	(38%)	-	
Severe	41	(18%)	-	
**Formal service use**				
Hired a foreign caregiver	74	(32%)	-	
Hired a domestic caregiver	12	(5%)	-	
Home care	15	(6%)	-	
Day care center	8	(3%)	-	
Short nursing home stay	6	(3%)	-	
***Caregiver characteristics***				
Age		-	61.0	13.2
Gender (% female)	146	(63%)	-	
Years in caregiving		-	4.8	3.1
Co-resident with patients	175	(76%)	-	
Primary caregiver	197	(85%)	-	
Employed	97	(42%)	-	
**Education**				
No education	12	(5%)	-	
Elementary school	34	(15%)	-	
Junior high	22	(10%)	-	
Senior high	82	(35%)	-	
College or above	81	(35%)	-	
**Relationship with the patients**				
Spouse	88	(38%)	-	
Adult children	109	(47%)	-	
Children-in-law	29	(13%)	-	
Grandchildren	5	(2%)	-	
**Economic status**				
Have difficulty	48	(21%)	-	
Have enough money.	147	(63%)	-	
Have more than enough money	36	(16%)	-	
**Informal care (hours/ week)**				
ADL		-	6.4	9.0
IADL		-	15.0	9.9
Supervision		-	27.2	33.2
Total hours		-	48.7	40.9

ADL = Activities of Daily Living; NPI = Neuropsychiatric Inventory.

Table 2 presents results of the bivariate analyses of annual total costs per person and detailed cost categories by severity of dementia. As severity increased, significant increases were found in the costs of social care, informal care, and total costs, but not in medical costs, for which a Chi-square test indicated no difference. Looking into medical costs by cost components, it was found that mild dementia patients actually had greater drug expenditures than either moderate or severe dementia patients. (We will come back to this seemingly unintuitive result in the Discussion section). Informal care cost estimates from both the opportunity costs and the replacement costs approach are listed in Table 2, with the first serving as the base-case estimate and the latter as an upper bound. The difference in the two estimation approaches also led to variations between total cost base-case estimates and total cost upper-bounds. According to the base-case estimate, the total costs for moderate dementia patients were 1.4 times the costs for mild dementia and doubled from mild to severe dementia. Despite the difference in absolute numbers, both sets of total cost estimates increased significantly by disease severity.

**Table 2:** Annual cost per person with dementia by disease severity.

Cost category	Mild (n = 102)		Moderate (n = 88)		Severe (n = 41)		P-value
	Mean NT$	SD	Mean NT$	SD	Mean NT$	SD	
Medical costs							
Outpatient	17,303	14,674	17,444	20,509	15,082	20,669	0.258
Drugs	30,065	35,229	25,630	24,427	13,767	9,892	0.002
Inpatient	14,228	45,470	11,591	52,251	8,820	34,480	0.251
OOP	24,093	28,146	27,118	24,831	38,378	41,886	0.195
Total	85,689	87,485	81,782	88,523	76,047	75,240	0.617
Social care costs	41,331	93,568	117,031	146,962	173,079	148,024	<0.001
Informal care costs							
Opportunity cost	91,623	140,151	110,134	131,133	190,846	202,290	0.006
Replacement cost	199,466	149,442	214,784	149,648	231,659	176,141	0.688
Total costs^a^							
Base-case	218,644	199,843	308,947	210,289	439,972	250,254	<0.001
Upper-bound	326,487	193,155	413,598	182,676	480,786	216,198	<0.001

OOP = out-of-pocket costs; 1NT = 14.97 US-PPP in 2014.

^a^ Total costs base-case estimate included informal care costs valued by the opportunity cost approach; total costs upper-bound included informal care costs valued by the replacement cost approach.

**Fig 1:**
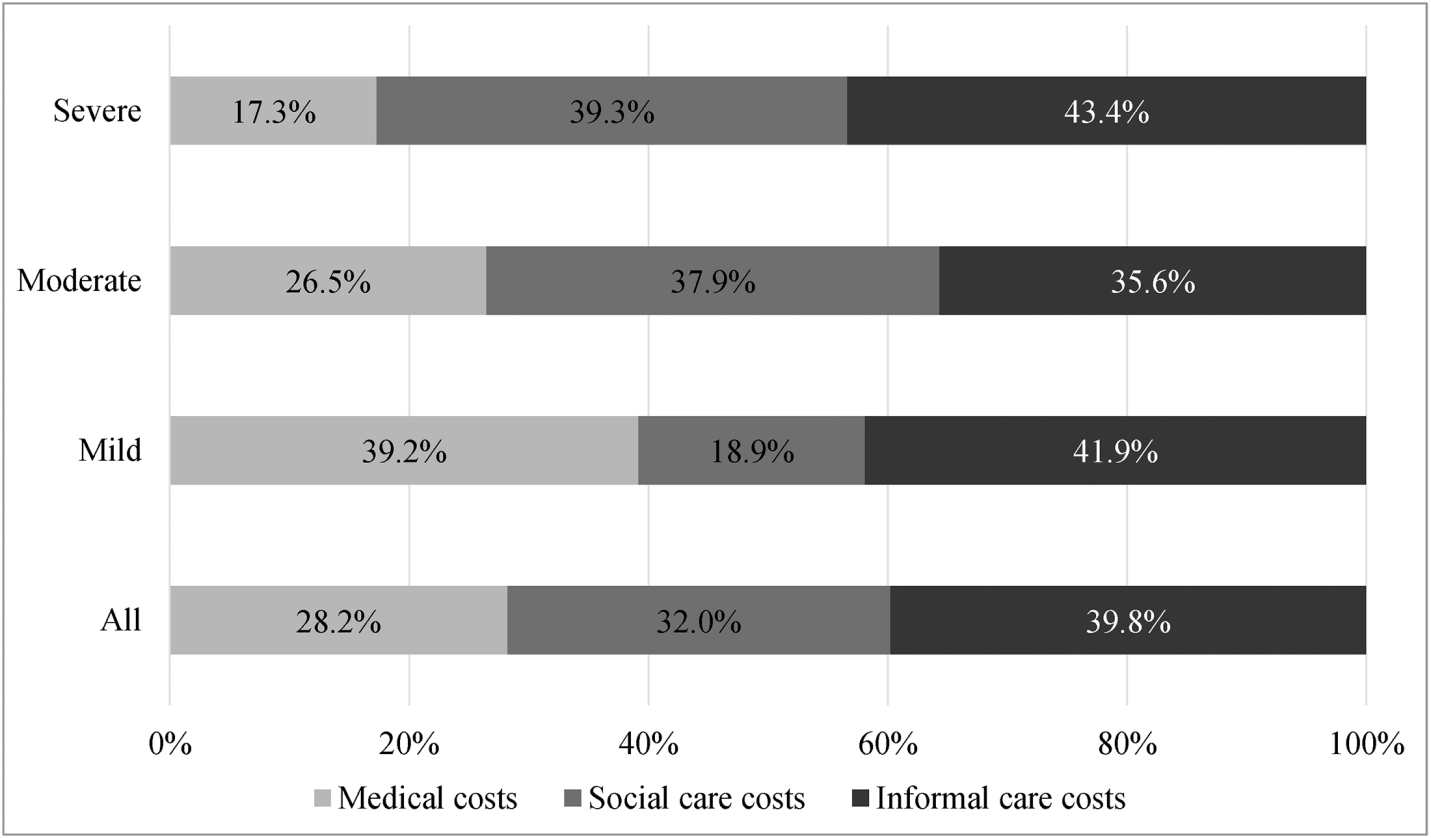
Proportion of medical, social care, and informal care cost in total cost per person with dementia by disease severity.

The proportion of medical, social care, and informal care cost in total cost per person with dementia is displayed in Fig 1. The bar chart highlights the fact that as dementia advances, social care costs increased strongly from mild to severe dementia while the relative importance of medical care as the cost driver decreased from 39.2% to 17.3%. Except for moderate dementia patients, informal care costs accounted for the greatest share in total cost of care for both mild and severe dementia patients. The last row in Fig 1 shows that in our full sample, regardless of severity, medical care contributed to only 28.2% of total care costs, while social care costs and informal care costs each accounted for 32% and 38.9% of the total costs, respectively.

Results of the GLM analysis (Table 3) indicated that in the multivariate analysis, functional declines measured by ADL had a greater impact on the cost of care than behavioral disturbance as measured by NPI across all four cost categories. Cognitive decline was not associated with higher costs except that informal care costs increased for severe dementia patients in comparison with mild dementia patients. Formal service use was associated with higher social care costs and total costs but lower informal care costs. Patients whose caregivers had completed senior high school and above or those with better economic status had greater total costs.

**Table 3:** Generalized linear models on costs of care (N = 231).

Variables	Medical care	Social care	Informal care	Total costs
***Patient characteristics***				
Age	-0.017	0.019	-0.030*	-0.007
	(0.012)	(0.011)	(0.013)	(0.007)
ADL score	-0.005*	-0.004*	-0.008***	-0.007***
	(0.002)	(0.002)	(0.002)	(0.001)
NPI score	-0.004	0.002	-0.000	-0.000
	(0.003)	(0.003)	(0.004)	(0.002)
Dementia severity (reference: mild)				
Moderate	-0.073	0.008	0.345	0.107
	(0.159)	(0.157)	(0.184)	(0.100)
Severe	-0.246	0.050	0.721*	0.196
	(0.185)	(0.170)	(0.288)	(0.132)
Formal service use (reference: no use)	0.128	4.604***	-0.764***	0.343***
	(0.168)	(0.398)	(0.169)	(0.100)
***Caregiver characteristics***				
Spousal CG (reference: non-spousal CG)	-0.104	0.145	-1.705***	-0.509***
	(0.183)	(0.126)	(0.360)	(0.125)
Senior high school and above	0.171	0.234	0.673*	0.350**
	(0.152)	(0.134)	(0.321)	(0.129)
Econ status (reference: Have enough money)				
Have plenty of money	0.489**	0.010	0.169	0.234*
	(0.161)	(0.119)	(0.234)	(0.107)
Have difficulty	-0.105	-0.656**	0.421*	-0.019
	(0.143)	(0.237)	(0.182)	(0.115)

Notes: All models assumed a log link function and a Poisson distribution after a Park’s test.

Standard errors in parentheses.

CG = Caregiver; ADL = Activities of Daily Living; NPI = Neuropsychiatric Inventory

* *p*<0.05.

** *p*<0.01.

*** *p*<0.001.

The marginal estimates of predicted cost differences by dementia severity are presented in Table 4. After adjusting for patient and caregiver characteristics, the model predicted that neither medical costs nor social care costs were significantly different for dementia patients at different stages. However, the informal care cost predictions were NT$122,363 for moderate and NT$178,141 for severe dementia, both being significantly higher than the NT$86,637 estimate for mild dementia.

**Table 4:** Marginal estimates of cost of care by dementia severity based on a GLM analysis adjusting for patient and caregiver variables ^a^.

Cost category	Mild (n = 102)	Moderate (n = 88)			Severe (n = 41)		
	Mean NT$	Mean NT$	Difference ^b^	P-value	Mean NT$	Difference ^c^	P-value
Medical costs	88,635	82,358	-6,276	0.64	69,280	-19,355	0.17
Social care costs	91,711	92,408	698	0.96	96,438	4,727	0.77
Informal care costs	86,637	122,363	35,726	0.06	178,141	91,504	0.03
Total costs	266,427	296,611	30,185	0.28	324,244	57,818	0.14

1NT = 14.97 US-PPP in 2014.

^a^ See Table 3 for patient and caregiver characteristics included in the generalized linear model (GLM).

^b^ Cost difference between moderate and mild patients.

^c^ Cost difference between severe and mild patients.

## Discussion

In this study, the annual COI of dementia in Taiwan was calculated, and the association between dementia severity and cost of care was examined through bivariate and multivariate analyses. In the bivariate analysis presented earlier, we found that total costs increased with dementia severity and that total costs doubled from mild to severe dementia, which was similar to a systematic review of COI studies of dementia, which reported that total costs more than doubled from mild to severe dementia [3]. However, our finding that mild dementia patients actually had greater drug expenditures than moderate or severe dementia patients was different from the results of earlier studies [8, 28]. The main reason for this is that Taiwan’s NHI reimbursement rule for dementia drugs stipulates that patients on cholinesterase inhibitors or memantine must be re-evaluated every year, and the drugs are no longer reimbursed following disease progression or abrupt deterioration [19]. A plausible reason why medical costs did not vary by dementia severity in our study was due to the nature of this disease—continuous support in daily living rather than curative treatment will naturally become more important as dementia progresses [8]. Since it was informal care rather than medical care that accounted for the majority of total costs of dementia and the costs of social care continued to rise with dementia severity, providing rehabilitation service such as physical and occupational therapy to dementia patients can be of great value to reduce dependencies that arises as the disease progresses. Although the annual costs of dementia were initially reported in NTD, the following PPP conversions for the base-case total cost estimates were US$14,609 (SD = + 13,353) for mild, US$20,643 (SD = + 14,051) for moderate, and US$29,398 (SD = +16,721) for severe dementia. A comparison of these numbers to the COI estimates reported in a systematic review of 8 studies examining dementia severity indicated that our mild dementia estimate was similar, but both our moderate and severe dementia estimates were much lower than those of previous studies (US$42,930 for moderate and US$51,659 for severe dementia). Given that all of the cost estimates were reported in community-based settings, we next looked into each cost category to try to explain the observed differences in costs.

Our results show that informal care taking the largest share of the total care costs is in line with the literature, in which informal costs were found to be the main cost driver [6]. However, in our study, the percentage of informal care to the total care costs was between 41.9% and 43.4%, while previous reviews have reported that across different stages, informal care usually contributes to more than half of the total costs of dementia among community samples [3, 5, 6]. Similar to previous studies, we found as dementia severity increases, so do social care costs, as does the proportion of social care to total costs [6, 8]. However, the overall share of social care costs (32%) was far less than the 43.1% reported among high income countries [4], where publicly-funded long-term care services are more available than in Taiwan. The greater contribution of informal care to total costs as opposed to social care also reflected traditional Asian family values, where family members of patients usually view caregiving as an obligation [7, 29].

By including measures of cognitive, functional, and behavioral disturbance in our multivariate model, we compared the strength of association between costs and different measures of disease severity. Similar to previous studies indicating that ADLs are highly correlated with both medical costs [30, 31] and informal care costs [32, 33], our results demonstrated ADLs to be associated with all cost categories, from medical costs to social care costs, as well as informal care costs. It is worth noting that the association between functional limitation and costs was significant regardless of other severity measures that were included. What we found regarding the null effect of behavioral disturbance on costs was similar to the conclusion of an earlier review on this topic [5]. On the other hand, it was somewhat surprising that the marginal effect of severe dementia was only significant in informal costs but not in other cost categories after adjusting for other disease indicators. In fact, the strength of the independent association between cognitive decline and cost of care remains questionable since some study has reported a positive association [28] while other studies has reported a null effect [31, 34]

Although the main objective of the current study was to examine the relationship between dementia severity and cost of care, in our regression analyses, we found that formal service use by dementia patients turned out to be an important predictor of care costs. One Singaporean study that calculated informal care costs related to caring for mild-moderate dementia patients in a community setting concluded that the use of paid domestic help resulted in cost savings [9]. Similar to that study, we found that formal service use was negatively associated with informal care costs. However, since our study found that formal service use was also correlated with higher social care costs and higher total costs, which implies that a substitution effect between formal services and informal care will still lead to higher societal costs overall [35].

There are several limitations in the current study we would like to acknowledge. First of all, our study sample came from a single dementia clinic in one university hospital in southern Taiwan with patients in a community setting, and thus, our COI estimates are not nationally representative and did not include patients living in institutions. Another limitation is that since the cognitive status of our sample came from their medical records, some of them were measured by the CDR but others by the MMSE only. However, it may be of concern that the CDR is a global measure of dementia and should not be equated with MMSE scoring. We therefore recommend that future studies use the CDR as a single measure of severity if such data is available. In fact, we also support the idea of calling for new research into a single measure that would capture three components of disease severity, including functional status and behavior disturbance, to ease the comparisons among different studies [5, 6].

## Conclusions

In conclusion, we found a positive relationship between dementia severity and the costs of caring for dementia in Taiwan, with a strong association between functional decline and higher total costs. This implies that the provision of rehabilitation intended to maintain patient physical function may reduce the overall cost of dementia. Given that informal care still accounts for the largest share of total costs, the government needs to promote long-term care services to increase the relative contribution of the social care sector related to assisting family caregivers of dementia patients.

## References

[1] Wimo A, Prince MJ. World Alzheimer Report 2010: the global economic impact of dementia: Alzheimer's Disease International; 2010.

[2] Alzheimer’s Association. 2015 Alzheimer's disease facts and figures. Alzheimer's & dementia: the journal of the Alzheimer's Association. 11(3):332. 10.1016/j.jalz.2015.02.003 25984581

[3] Quentin W, Riedel-Heller SG, Luppa M, Rudolph A, Konig HH. Cost-of-illness studies of dementia: a systematic review focusing on stage dependency of costs. Acta psychiatrica Scandinavica. 2010;121(4):243–59. 10.1111/j.1600-0447.2009.01461.x . 19694634

[4] Wimo A, Prince MJ, Guerchet M, Ali G-C, Wu Y-T, Prina M. World Alzheimer Report 2015: the global impact of dementia. Alzheimer's Disease International, 2015.

[5] Mauskopf J, Racketa J, Sherrill E. Alzheimer's disease: the strength of association of costs with different measures of disease severity. The journal of nutrition, health & aging. 2010;14(8):655–63. Epub 2010/10/06. . 20922342 10.1007/s12603-010-0312-6 PMC12880306

[6] Schaller S, Mauskopf J, Kriza C, Wahlster P, Kolominsky‐Rabas PL. The main cost drivers in dementia: a systematic review. International journal of geriatric psychiatry. 2015;30(2):111–29. 10.1002/gps.4198 25320002

[7] Lim J, Goh J, Chionh HL, Yap P. Why do patients and their families not use services for dementia? Perspectives from a developed Asian country. International Psychogeriatrics. 2012;24(10):1571–80. 10.1017/S1041610212000919 22647248

[8] Schwarzkopf L, Menn P, Kunz S, Holle R, Lauterberg J, Marx P, et al Costs of care for dementia patients in community setting: an analysis for mild and moderate disease stage. Value Health. 2011;14(6):827–35. Epub 2011/09/15. 10.1016/j.jval.2011.04.005 . 21914502

[9] Chong MS, Tan WS, Chan M, Lim WS, Ali N, Ang YY, et al Cost of informal care for community-dwelling mild–moderate dementia patients in a developed Southeast Asian country. International psychogeriatrics. 2013;25(09):1475–83. 10.1017/S1041610213000707 23694676

[10] Fuh JL, Wang SJ. Dementia in Taiwan: past, present, and future. Acta Neurologica Taiwanica. 2008;17(3):153–61. 18975520

[11] Sun Y, Lee HJ, Yang SC, Chen TF, Lin KN, Lin CC, et al A nationwide survey of mild cognitive impairment and dementia, including very mild dementia, in Taiwan. PLOS one. 2014;9(6):e100303 Epub 2014/06/19. 10.1371/journal.pone.0100303 24940604 PMC4062510

[12] Chou LF, Chang CW, Fu JL, Wang SJ. The economic costs of dementia in Taiwan. Natil Chengchi Univ J. 2000;82:1–26.

[13] Kuo YC, Lan CF, Chen LK, Lan VM. Dementia care costs and the patient's quality of life (QoL) in Taiwan: home versus institutional care services. Archives of gerontology and geriatrics. 2010;51(2):159–63. Epub 2010/01/01. 10.1016/j.archger.2009.10.001 . 20042244

[14] Jonsson L, Wimo A. The cost of dementia in Europe: a review of the evidence, and methodological considerations. PharmacoEconomics. 2009;27(5):391–403. 10.2165/00019053-200927050-00004 19586077

[15] Rapp T, Andrieu S, Molinier L, Grand A, Cantet C, Mullins CD, et al Exploring the relationship between Alzheimer's disease severity and longitudinal costs. Value in health: the journal of the International Society for Pharmacoeconomics and Outcomes Research. 2012;15(3):412–9. 10.1016/j.jval.2012.02.003 . 22583450

[16] American Psychiatric Association. Diagnostic and Statistical Manual of Mental Disorders, Fourth Edition 1994.

[17] Hughes CP, Berg L, Danziger WL, Coben LA, Martin RL. A new clinical scale for the staging of dementia. The British Journal of Psychiatry. 1982;140(6):566–72. 7104545 10.1192/bjp.140.6.566

[18] Chuo LJ, Sheu WHH, Pai MC, Kuo YM. Genotype and Plasma Concentration of Cystatin C in Patients with Late-Onset Alzheimer Disease. Dementia and Geriatric Cognitive Disorders. 2007;23(4):251–7. 17310123 10.1159/000100021

[19] Bureau of National Health Insurance. Reimbursement rule of drugs acting on the nervous system Taiwan 2013. Available: http://www.nhi.gov.tw/. Accessed 9 July 2013.

[20] Benaim C, Froger J, Compan B, Pelissier J. The assessment of autonomy in elderly people. Ann Readapt Med Phys. 2005;48(6):336–40. 15932780 10.1016/j.annrmp.2005.04.005

[21] Thomas P, Lalloué F, Preux PM, Hazif‐Thomas C, Pariel S, Inscale R, et al Dementia patients caregivers quality of life: the PIXEL study. International journal of geriatric psychiatry. 2006;21(1):50–6. 16323256 10.1002/gps.1422

[22] Cummings JL, Mega M, Gray K, Rosenberg-Thompson S, Carusi DA, Gornbein J. The Neuropsychiatric Inventory comprehensive assessment of psychopathology in dementia. Neurology. 1994;44(12):2308- 7991117 10.1212/wnl.44.12.2308

[23] Wimo A, Gustavsson A, Jonsson L, Winblad B, Hsu MA, Gannon B. Application of Resource Utilization in Dementia (RUD) instrument in a global setting. Alzheimer's & dementia: the journal of the Alzheimer's Association. 2013;9(4):429–35 e17. Epub 2012/11/13. 10.1016/j.jalz.2012.06.008 . 23142433

[24] Ministry of Health and Welfare. Social Welfare Subsidized Items and Schedule 2014 [updated 2014/09/24]. Available: http://www.sfaa.gov.tw/SFAA/Pages/Detail.aspx?nodeid=428&pid=3156.

[25] International Monetary Fund. World Economic Outlook Database 2015. Available: https://www.imf.org/external/pubs/ft/weo/2015/01/weodata/index.aspx.

[26] Manning WG, Mullahy J. Estimating log models: to transform or not to transform? Journal of health economics. 2001;20(4):461–94. 11469231 10.1016/s0167-6296(01)00086-8

[27] Lafortune L, Béland F, Bergman H, Ankri J. Health state profiles and service utilization in community-living elderly. Medical care. 2009;47(3):286–94. 10.1097/MLR.0b013e3181894293 19165121

[28] Jönsson L, Jönhagen ME, Kilander L, Soininen H, Hallikainen M, Waldemar G, et al Determinants of costs of care for patients with Alzheimer's disease. International journal of geriatric psychiatry. 2006;21(5):449–59. 16676288 10.1002/gps.1489

[29] Chan SWC. Family Caregiving in Dementia: The Asian Perspective of a Global Problem. Dementia and Geriatric Cognitive Disorders. 2010;30(6):469–78. 10.1159/000322086 WOS:000286429200005. 21252540

[30] Hill J, F H, Thomas SK, Chang S. Functional impairment, healthcare costs and the prevalence of institutionalization in patients with Alzheimer's disease and other dementias. Pharmacoeconomics. 2006;24(3):265–80. 16519548 10.2165/00019053-200624030-00006

[31] Leicht H, Konig HH, Stuhldreher N, Bachmann C, Bickel H, Fuchs A, et al Predictors of costs in dementia in a longitudinal perspective. PLOS One. 2013;8(7):e70018 10.1371/journal.pone.0070018 23875017 PMC3715502

[32] Small GW, M D, Brooks RL, Papadopoulos G. The impact of symptom severity on the cost of Alzheimer's disease. Journal of the American Geriatrics Society. 2002;50(2):321–7. 12028215 10.1046/j.1532-5415.2002.50065.x

[33] Neubauer S, Holle R, Menn P, Grossfeld-Schmitz M, Graesel E. Measurement of informal care time in a study of patients with dementia. International psychogeriatrics / IPA. 2008;20(6):1160–76. 10.1017/S1041610208007564 . 18606044

[34] Zhu CW, Leibman C, McLaughlin T, Zbrozek AS, Scarmeas N, Albert M, et al Patient dependence and longitudinal changes in costs of care in Alzheimer's disease. Dementia and Geriatric Cognitive Disorders. 2008;26(5):416–23. Epub 2008/10/24. 10.1159/000164797 18946219 PMC2631662

[35] Gerves C, Chauvin P, Bellanger MM. Evaluation of full costs of care for patients with Alzheimer's disease in France: the predominant role of informal care. Health Policy. 2014;116(1):114–22. 10.1016/j.healthpol.2014.01.001 . 24461717

